# Osimertinib-induced interstitial lung disease after treatment with anti-PD1 antibody

**DOI:** 10.1007/s10637-016-0389-9

**Published:** 2016-09-06

**Authors:** Nobuaki Mamesaya, Hirotsugu Kenmotsu, Mineo Katsumata, Takashi Nakajima, Masahiro Endo, Toshiaki Takahashi

**Affiliations:** 1 Division of Thoracic Oncology, Shizuoka Cancer Center Hospital, 1007 Shimonagakubo, Nagaizumi-cho, Sunto-gun, Shizuoka, 411-8777 Japan; 2 Department of Pulmonary Medicine, Seirei Hamamatsu General Hospital, Shizuoka, Japan; 3 Division of Pathology, Shizuoka Cancer Center Hospital, Shizuoka, Japan; 4 Division of Diagnostic Radiology, Shizuoka Cancer Center Hospital, Shizuoka, Japan

**Keywords:** Lung cancer, *EGFR* mutation, *EGFR* tyrosine kinase inhibitor, Anti-PD1 antibody, Interstitial lung disease

## Abstract

We report a case of a 38-year-old woman who was diagnosed with stage IV lung adenocarcinoma, harboring an epidermal growth factor receptor (*EGFR*) L858R mutation on exon 21 and a T790 M mutation on exon 20. The patient was treated with osimertinib, a third-generation *EGFR* tyrosine kinase inhibitor (*EGFR*-TKI) following treatment with nivolumab, an anti-Programmed Cell Death 1 (anti-PD1) antibody. After initiating osimertinib treatment, the patient began to complain of low-grade fever and shortness of breath without hypoxemia, and her chest radiograph and a CT scan revealed a remarkable antitumor response, although faint infiltrations were observed in the bilateral lung field. Bronchoalveolar lavage fluid mainly contained lymphocytes (CD4+/CD8+ ratio of 0.3), and a transbronchial lung biopsy specimen showed lymphocytic alveolitis with partial organization in several alveolar spaces. Therefore we diagnosed the patient with osimertinib-induced interstitial lung disease (ILD) after treatment with anti-PD1 antibody. We considered anti-PD1 therapies may be the risk factor of *EGFR*-TKI-induced ILD.

## Case report

A 38-year-old woman with no smoking history noticed right shoulder pain and dyspnea on exertion in December 2014, and presented at hospital in January 2015. She was diagnosed with stage IV lung adenocarcinoma, harboring an epidermal growth factor receptor (*EGFR*) L858R mutation on exon 21. She was treated with bevacizumab plus erlotinib as first-line chemotherapy, and subsequently received carboplatin plus pemetrexed. In February 2016, nivolumab at a dose of 3 mg/kg was administered every 2 weeks up to four cycles. However, no significant response was observed, and a CT-guided needle biopsy of the pleural lesion was performed to evaluate *EGFR* mutation status. The biopsy specimens revealed adenocarcinoma harboring an *EGFR* L858R mutation on exon 21 and a T790 M mutation on exon 20. The patient was referred to our institution in April 2016 to be treated with osimertinib, a third-generation *EGFR* tyrosine kinase inhibitor (*EGFR*-TKI). As the tumor had shown dramatic and aggressive growth since diagnosis, osimertinib treatment (80 mg, once daily) was initiated 8 days after the last administration of nivolumab.

**Fig. 1:**
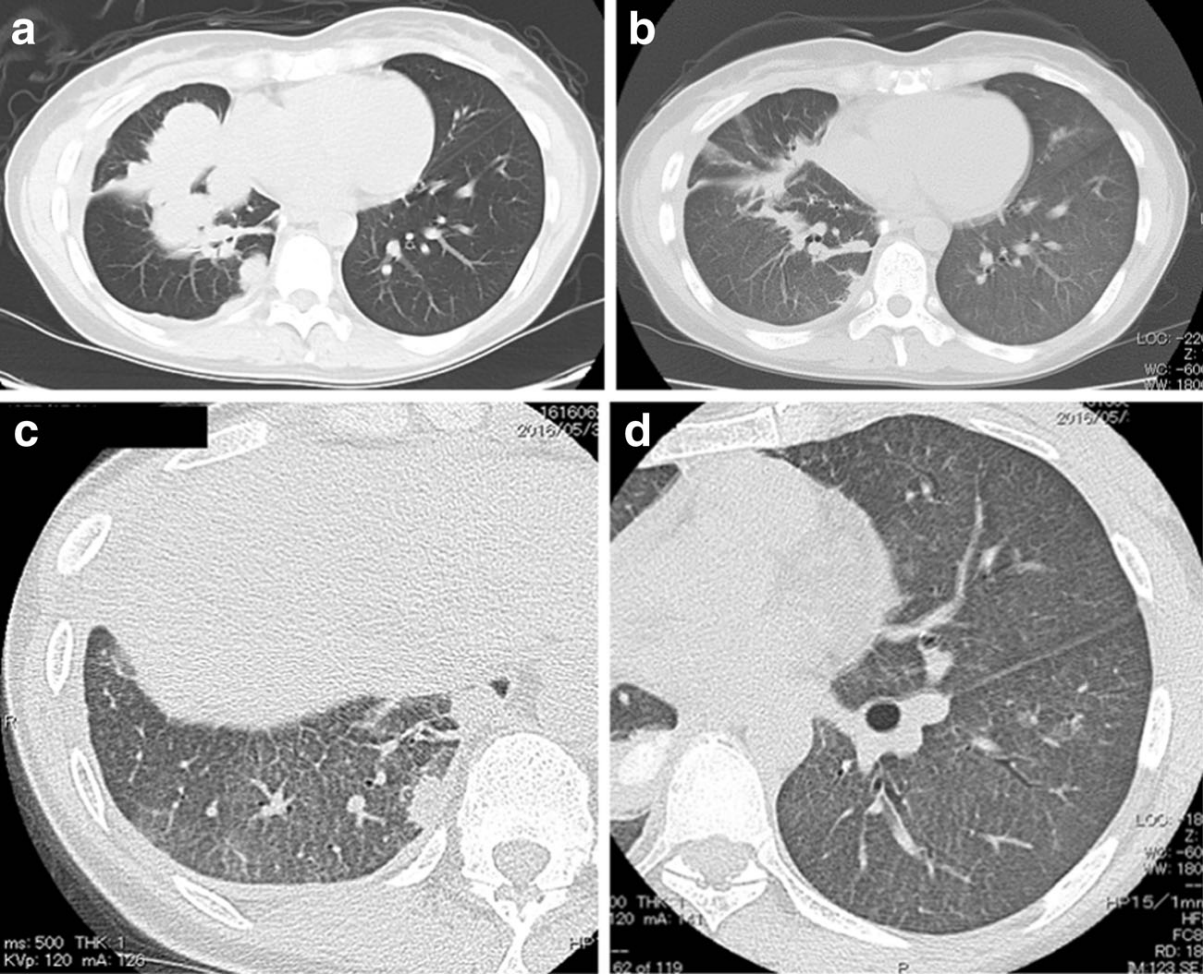
Chest CT findings before and after treatment. **a** Solid tumors with intrapulmonary metastasis and no evidence of preexisting interstitial pneumonia before treatment with osimertinib. **b** Remarkable shrinkage of tumors after treatment with osimertinib. **c**, **d** Diffuse, faint, ground-glass opacities lacking consolidation and traction bronchiectasis in both lungs after treatment with osimertinib

Thirty-one days after initiating osimertinib treatment, the patient began to complain of low-grade fever and shortness of breath without hypoxemia. Her chest radiograph revealed a remarkable antitumor response with an improvement in right pleural thickening and pleural effusion, although faint infiltrations were observed in the bilateral lung field. Because the possibility of osimertinib-induced interstitial lung disease (ILD) was considered, treatment with osimertinib was discontinued. After osimertinib treatment interruption, the patient’s symptoms were gradually relieved and osimertinib was re-administered following consideration of the clinical benefit of its antitumor efficacy. A week after resumption of osimertinib treatment, however, her symptoms worsened and bilateral faint shadows were observed by chest radiograph. Furthermore, the chest CT scan revealed further tumor shrinkage and bilateral diffuse, faint, ground-glass opacities lacking consolidation and traction bronchiectasis, a pattern compatible with hypersensitivity pneumonitis (Fig. 1). Thus, osimertinib-induced ILD was strongly suspected and we performed various appropriate examinations.

**Fig. 2:**
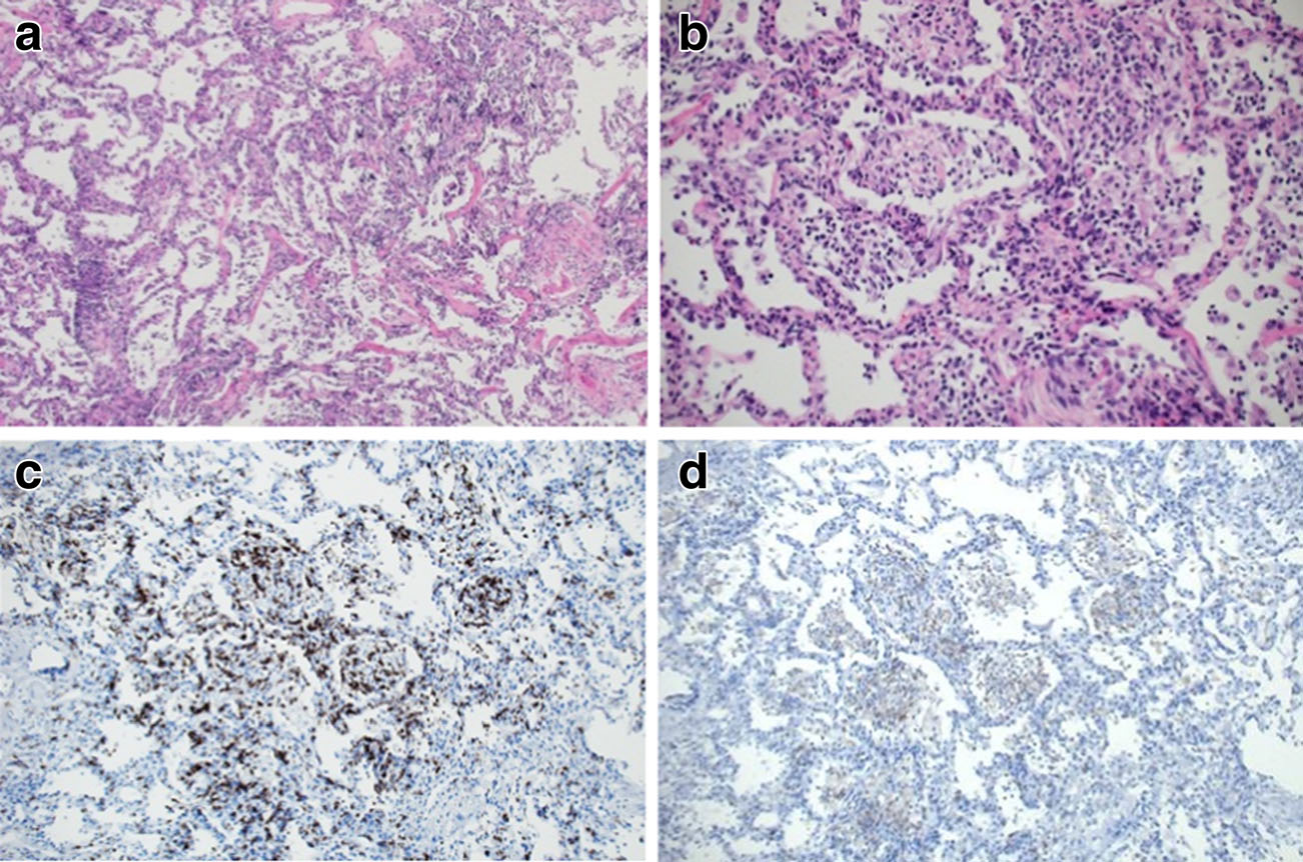
Transbronchial lung biopsy specimen following treatment with osimertinib. **a**, **b** Lymphocytic alveolitis with partial organization (HE staining at low- and high-powered fields). **c** Strong CD8+ cytotoxic T-cell lymphocyte-positive infiltrate was mainly distributed in the alveolar septum (CD8 immunostaining at high-powered field). **d** Weak CD4+ helper T-cell lymphocyte-positive infiltrate mainly distributed in the alveolar septum (CD4 immunostaining at high-powered field)

Bronchoalveolar lavage fluid (BALF), obtained from the left bronchus (90 mL/150 mL), contained a white blood count of 1660 cells/μL (83 % lymphocytes, 14.5 % macrophages, 1 % neutrophils, and 1 % eosinophils) and showed a CD4+/CD8+ ratio of 0.3. BALF culture was negative for pathogens including bacteria, fungi, acid-fast bacilli, and *pneumocystis jirovecii* by polymerase chain reaction. A transbronchial lung biopsy specimen showed mainly lymphocytic alveolitis with partial organization in several alveolar spaces, without severe fibrosis. There was no evidence of infection, cardiogenic pulmonary edema, carcinomatous lymphangitis, pulmonary hemorrhage, or hyaline membranes (Fig. 2). This pathological pattern was compatible with previous reports of drug-induced ILD, although there are few reports on the specific pathological features of *EGFR*-TKI-induced ILD [1]. Although general blood tests including serum inflammatory markers, white blood cell count, and CRP level were within the normal range, serum KL-6 was elevated from 547 U/mL (May 2016) to 2310 U/mL (June 2016). Based on these clinical findings, we diagnosed the patient with osimertinib-induced ILD. Following withdrawal of osimertinib, her symptoms and the faint shadows on the bilateral lung field gradually diminished without administration of steroids, further supporting the diagnosis of osimertinib-induced ILD. Two months after discontinuation of osimertinib treatment, a chest CT scan showed that the bilateral diffuse, faint, ground-glass opacities had disappeared completely, but right pleural thickening had worsened. Therefore, she has been treated with ramucirumab plus docetaxel as subsequent chemotherapy from August 2016.

## Discussion

To our knowledge, this is the first case report of pathologically proven lung injury caused by osimertinib. Osimertinib is effective against both *EGFR*-sensitizing and T790 M mutations, and has recently been approved in Japan. Incidence of osimertinib-induced ILD was reported as 3 %, and grade 5 was observed in 1 % of cases [2]. However, the incidence of anti-Programmed Cell Death 1 (anti-PD1) antibody-induced pulmonary toxicities has been reported as 9 %, with grade 5 observed in 2 % of cases [3]. Recently, updated results from the osimertinib plus durvalumab (MEDI4736) anti-Programmed Cell Death 1 Ligand 1 monoclonal antibody combination arm of the TATTON study (NCT02143466) have been reported [4]. ILD was observed in 38 % of patients treated with the combination of osimertinib plus durvalumab. Anti-PD1 therapies may potentially enhance immunological reactions related to CD8+ cytotoxic T-cell lymphocyte, including osimertinib-induced ILD. In this case, nivolumab administered 1 week before treatment with osimertinib was considered to be a possible risk factor for *EGFR*-TKI-induced ILD.

In conclusion, we report radiological and pathological findings of osimertinib-induced ILD following treatment with an anti-PD1 antibody. The possibility of increasing the risk of *EGFR*-TKI-induced ILD after anti-PD1 antibody therapy therefore exists. Osimertinib-induced ILD has been previously reported and can be associated with fatal adverse events. Careful monitoring of the patient’s clinical course for any relevant symptoms to ensure early detection of ILD may be important to prevent the development of life-threatening ILD. Further studies are warranted, however, to clarify the risk factors for osimertinib-induced ILD.
